# The Board of Health and Dr. Olmsted’s Bill

**Published:** 1898-02

**Authors:** 


### THE BOARD OF HEALTH AND DR. OLMSTED'S BILL.

When Atlanta opened her gates to the refugees from the late yellow fever scare along the gulf coast, she admitted two persons, already infected, who went through a mild course of the disease here. Recognizing the importance of having these patients well cared for, the Board of Health engaged the services of physicians to attend them who were known to have had experience in the treatment of yellow fever. One of these gentlemen was Dr. John C. Olmsted, and to his care one of the patients, a girl from Mobile, was entirely committed. He was also called upon to make some visits to suspicious cases and to the other patient above referred to. Dr. Olmsted was in attendance upon his single patient for a month, visiting her two and three times per day, and sometimes at night; portions of the time he was both physician and nurse, and he spared neither pains nor trouble in his management of the case. During this time his usual practice was almost entirely abandoned; or rather, his practice abandoned him. The patient recovered, and the city escaped the unwelcome consequences which would have followed a death from the fever.

Dr. Olmsted estimated his services to the city to be worth five hundred dollars, and presented a bill to the Board of Health for that amount.

The Board was then composed of five physicians, one lawyer, one business man, and the mayor (*ex officio*). By a vote of five to one payment of the bill was declined, on the ground that it was excessive (the President of the Board did not vote, though he approved the bill). The Board was willing to settle with Dr. Olmsted for three hundred dollars, which the doctor promptly refused.

Thus the matter stands at this writing. The action of the Board has occasioned no little surprise in the profession and even among the laity. Without questioning the honest convictions of the individual members of the Board of Health in voting to withhold payment of this bill, and while fully sympathizing with the Board in its desire to economize the public funds, we do not consider the compensation asked by Dr. Olmsted for his services in this somewhat extraordinary case to be at all exorbitant, and we do not be lieve that the majority of our well-informed citizens would object to its being paid. In our opinion the bill was reasonable and should have been allowed without hesitation.

Since the above was written some changes have taken place in the *personnel* of the Board, and it is thought that the new Board will approve the bill.

---

A QUARANTINE convention of the South Atlantic and Gulf States will be held in Mobile about February 1st, for the purpose of studying in an exhaustive way the theory and practice of quarantine, and to adopt some measures whereby the recent embarrassing situations in the South resulting from the late epidemic may be averted in the future. The call has been issued by the State Health Officer of Alabama. Delegates will be apportioned according to the population of the city from which they come. Each city with 10,000 inhabitants will be entitled to one delegate and one delegate more for every additional 3,000 inhabitants which the city may possess. It is hoped that the convention may be productive of much real good.

---

THE Atlanta Society of Medicine will meet the first and third Thursday nights in each month, instead of Tuesday nights as heretofore.

